# Diagnosis of Septic Abortion with Point-of-care Ultrasound

**DOI:** 10.5811/cpcem.2017.3.33574

**Published:** 2017-07-06

**Authors:** Dawa Sherpa, Brian D. Johnson, Leila Ben-Youssef, Arun Nagdev

**Affiliations:** * Alameda Health System – Highland Hospital, Oakland, California; † University of Washington – Valley Medical Center, Renton, Washington; ‡ University of California, San Francisco, San Francisco, California

## CASE PRESENTATION

A 19-year-old recently immigrated female presented with severe lower abdominal pain, fever, and vaginal bleeding after a syncopal episode. On examination, she was febrile with diffuse tenderness to palpation of her lower abdomen with an enlarged uterus palpable to just below the umbilicus. The patient initially denied pregnancy, but had a positive urine pregnancy with a quantitative beta-human chorionic gonadotropin of 19,773 mIU/mL. Pelvic exam revealed scant amount of blood in the vaginal canal with malodorous discharge. A point-of-care ultrasound revealed an enlarged uterus with a large amount of air and echogenic debris within the endometrium (Image). Over the course of the emergency department (ED) stay, the patient became hypotensive and was found to have anemia and leukocytosis requiring a blood transfusion, antibiotics and emergent gynecological intervention.

## DISCUSSION

The patient was admitted to the obstetric service and started on broad-spectrum antibiotics for incomplete septic abortion. She underwent a vacuum-assisted dilation and curettage with removal of uterine contents. Surgical pathology revealed degenerating and focal necrotic chorionic villi consistent with intra-uterine pregnancy. While rare in developed countries, septic abortion is a life-threatening infection of the placenta and fetus of a previable pregnancy typically associated with unsafe abortion practices.^1^ Diagnosis is made clinically and confirmed by ultrasound, computed tomography or magnetic resonance imaging that can show an enlarged uterus with hemorrhage, retained intrauterine material, free fluid, abscess formation and/or air. 2 Intrauterine air is theorized to arise from gas-forming organisms or secondary to perforation from unsafe abortion practices. The ultrasonographic finding of air in the uterus must be recognized by the emergency physician in order to expedite ED and consultative care. With the reduction in funding for healthcare services for at-risk populations and possible defunding of Planned Parenthood, we believe that this uncommon finding may become more prevalent for providers on the front line.^4^,^5^ Treatment consists of broad-spectrum antibiotics, prompt removal of infected tissue and hysterectomy in severe, refractory cases.^3^

CPC-EM Capsule

What do we already know about this clinical entity?

Septic abortion is a life-threatening infection associated with unsafe abortion practices that is diagnosed clinically and confirmed with ultrasound.

What is the major impact of the image(s)?

Our ultrasound video demonstrates septic abortion-associated uterine free air, a novel finding that has yet to be discussed in the point-of-care ultrasound (POCUS) emergency medicine literature.

How might this improve emergency medicine practice?

POCUS allows for prompt identification of septic abortion and swift consultation with obstetrics for definitive care.

## Supplementary Information

Video Transabdominal ultrasound of the uterus with debris and air with shadowing artifact.

## Figures and Tables

**Image:**
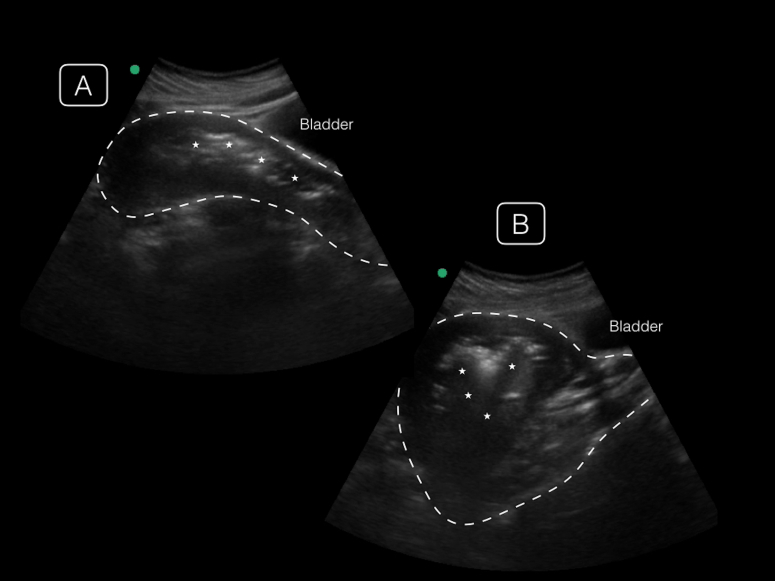
Point-of-care ultrasonographic transabdominal images of the uterus in midline (A) and off-axis sagittal views (B), demonstrating an enlarged uterus (dotted line) with irregular echogenic endometrial debris (asterisk) casting shadows from the endometrium.
